# Atomic imaging of mechanically induced topological transition of ferroelectric vortices

**DOI:** 10.1038/s41467-020-15616-y

**Published:** 2020-04-15

**Authors:** Pan Chen, Xiangli Zhong, Jacob A. Zorn, Mingqiang Li, Yuanwei Sun, Adeel Y. Abid, Chuanlai Ren, Yuehui Li, Xiaomei Li, Xiumei Ma, Jinbin Wang, Kaihui Liu, Zhi Xu, Congbing Tan, Longqing Chen, Peng Gao, Xuedong Bai

**Affiliations:** 1 Beijing National Laboratory for Condensed Matter Physics, Institute of Physics, Chinese Academy of Sciences, Beijing, 100190 China; 2 School of Physical Sciences, University of Chinese Academy of Sciences, Beijing, 100190 China; 3 School of Materials Science and Engineering, Xiangtan University, Hunan, Xiangtan 411105 China; 4 Department of Materials Science and Engineering, Penn State University, University Park, PA 16802 USA; 5 Electron microscopy laboratory, School of Physics, Peking University, Beijing, 100871 China; 6 International Center for Quantum Materials, Peking University, Beijing, 100871 China; 7 State Key Laboratory for Artificial Microstructure & Mesoscopic Physics, School of Physics, Peking University, Beijing, 100871 China; 8 Collaborative Innovation Center of Quantum Matter, Beijing, 100871 China; 9 Songshan Lake Materials Laboratory, Dongguan, Guangdong 523808 China; 10 Department of Physics and Electronic Science, Hunan University of Science and Technology, Hunan, Xiangtan 411201 China

**Keywords:** Ferroelectrics and multiferroics, Surfaces, interfaces and thin films

## Abstract

Ferroelectric vortices formed through complex lattice–charge interactions have great potential in applications for future nanoelectronics such as memories. For practical applications, it is crucial to manipulate these topological states under external stimuli. Here, we apply mechanical loads to locally manipulate the vortices in a PbTiO_3_/SrTiO_3_ superlattice via atomically resolved in-situ scanning transmission electron microscopy. The vortices undergo a transition to the *a*-domain with in-plane polarization under external compressive stress and spontaneously recover after removal of the stress. We reveal the detailed transition process at the atomic scale and reproduce this numerically using phase-field simulations. These findings provide new pathways to control the exotic topological ferroelectric structures for future nanoelectronics and also valuable insights into understanding of lattice-charge interactions at nanoscale.

## Abstract (web summary)

Controlling topological polar vortices promises to open up new applications for ferroelectric materials. Here, the authors proposed a method to mechanically manipulate polar vortices and monitored the transition between vortex and ferroelectric phase by in-situ scanning transmission electron microscopy.

## Introduction

Ferroelectric vortices^1–4^ composed of electric dipole moments with continuous rotation are theoretically predicted to occur in nanostructures such as nanowires^5^, nanodots^6^, and nanocomposites^7^, and have been experimentally realized in rhombohedral BiFeO_3_^8–11^, tetragonal PbZr_0.2_Ti_0.8_O_3_^12^ thin films, and PbTiO_3_/SrTiO_3_ (PTO/STO) superlattices^3^. These polarization vortex states that are regarded as topological defects and can be characterized by an electric toroidal moment, can exist over a few nanometers, making them a perfect candidate for data-storage applications^13^, as their storage capacity can reach ~10^12^ bits per square inch with little cross-talk between adjacent bits, which is several orders of magnitude larger than the current technology of ferroelectric storages^1^.

To enable the application of the polarization vortices in the systems described above in nanotechnology, we need to be able to effectively manipulate the order parameters and phase transitions under external stimuli. Theoretical studies have proposed switching either by a curled electric field^14^ or by rationally designed nanostructures, such as nanorings^15^ or notched nanodots^16^. Experimental investigation was only limited in a phase coexistence (vortex and *a*_1_/*a*_2_ phase) system by Damodaran et al.^17^, who used an atomic force microscopy (AFM) tip to apply an external electric field to a mixed-phase system, and demonstrated the interconversion between vortices and the regular ferroelectric phase by X-ray diffraction and second-harmonic generation. However, the knowledge of polarization evolution of a single vortex, such as nucleation, propagation, and stability during phase transition, remains largely unknown due to the difficulties in characterizing the small size of the vortices (typically 2–4 nm) in the buried film by the surface probe, X-ray diffraction, or optical detection. Hence, a novel route that possesses higher spatial resolution in structural characterization is highly desired.

Herein, we report a method based on local mechanical loading as an alternative stimulus to control vortices by making use of the intrinsic coupling between the strain and polarization^18^. Although previously such a mechanical stress method has been employed to switch the ferroelastic domains in thin films^19,20^, it has never been employed for manipulating the ferroelectric vortices because of the difficulties in fabrication of the vortex array and polarization characterization at nanometer scale. We combine atomically resolved in situ transmission electron microscopy (TEM) and phase-field modeling methods to study the behavior of the polar vortices in PTO/STO superlattices under mechanical stress. In contrast to conventional substrate-mediated strain control, in situ TEM can exert a continuously adjustable stress to a specimen by controlling the indenter freely and allow for real-time observation of the entire switching process, enabling dynamic behavior to be correlated with the applied excitations^20,21^. In this work, using the custom-built in situ TEM holder with much improved stability and carrying out the experiments in a spherical aberration-corrected TEM with subangstrom resolution allows us to directly monitor the evolution of each vortex and further map the local lattice and polarization during manipulating it by external excitations.

We find that the polar vortex transforms into the *a* domain with in-plane polarization under a compressive strain, and spontaneously recovers to the original vortex state after removal of the stress, demonstrating the reversibility of controlling the vortices by mechanical force. Such a phase transition occurs through the inhomogeneous nucleation and growth of newly formed *a* domains, which is confirmed by the selected-area electron diffraction (SAED) pattern and atomically resolved high-angle annular dark-field scanning transmission electron microscopy (HAADF-STEM) images. Phase-field modeling precisely reproduces such switching events. The single-vortex evolution and the stability of vortices are also discussed. These results provide valuable insights into understanding how the mechanical boundary conditions determine the polar states in PTO/STO superlattices. The demonstrated ability to manipulate the ferroelectric vortices at the atomic scale by mechanical stimuli may be valuable for the design of novel electromechanical nanoscale ferroelectric devices.

## Results

### Characterization of the vortex array in PTO/STO superlattice

**Fig. 1:**
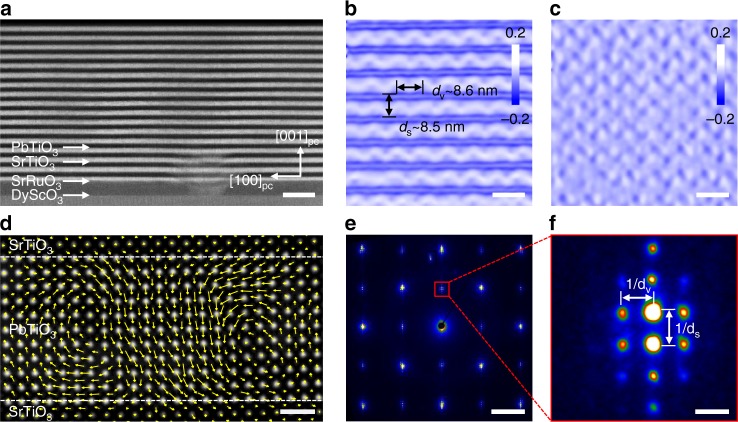
Characterization of vortices in PbTiO_3_/SrTiO_3_ superlattices. **a** Low-magnification scanning transmission electron microscopy (STEM) image of a (PbTiO_3_)_11_/(SrTiO_3_)_11_ superlattice along [010]_pc_, showing the alternative arrangement of SrTiO_3_ and PbTiO_3_ on a DyScO_3_ substrate. Scale bar, 25 nm. **b**, **c** Geometric-phase analysis of the STEM data showing the distribution of the out-of-plane strain *Ɛ*_*zz*_ and in-plane strain *Ɛ*_*xx*_, respectively. Scale bar, 10 nm. **d** Cross-sectional high-angle annular dark-field (HAADF) STEM image with an overlay of the polar displacement vectors denoted by the yellow arrows showing the vortices in the PbTiO_3_ layer. Scale bar, 1 nm. **e** A selected-area electron diffraction (SAED) pattern for the PbTiO_3_/SrTiO_3_ film. Scale bar, 2 nm^−1^. **f** Enlarged (001) spots showing the satellite diffraction spots from the ordered vortex. The vortex and superlattice periods are measured to be 8.6 and 8.5 nm, respectively, denoted by *d*_v_ and *d*_s_. Scale bar, 0.1 nm^−1^.

Thin (PTO)_n_/(STO)_*n*_ (*n* denotes the thickness of STO and PTO in terms of the number of unit cells) films were grown on (110)_o_ DyScO_3_ substrates with a SrRuO_3_ (SRO) buffer layer by pulsed-laser deposition (subscript o denotes orthorhombic structure). The experimental details are presented in the “Methods” section. Figure 1a shows a low-magnification atomically resolved HAADF image of a (PTO)_11_/(STO)_11_ film, where the bright and dark contrast indicates the PTO and STO layers, respectively. The sinusoidal array of out-of-plane and in-plane strain, which is easily distinguished by geometric-phase analysis (GPA) of the HAADF images in Fig. 1b, c, is indicative of long-range ordered arrays of the domain structures. Figure 1d shows a polarization map of the PTO layer obtained by calculating the offsets between the Pb and Ti sublattices^22,23^, with the overlaid vectors representing the magnitude and direction of the polar displacements. As a result, the polarization exhibits a continuous rotation, resulting in a vortex structure similar to that reported in a previous study^3^. A SAED pattern acquired from a region that only includes the PTO/STO superlattice layers is shown in Fig. 1e. The equally spaced spots in the out-of-plane direction reflect the long-range order of the superlattices. Close inspection reveals another set of reflections along the in-plane direction similar to that observed from X-ray diffraction pattern^3,17^, indicating long-range ordering of vortices in each layer. The in-plane periodicity of the vortices is ~8.6 nm in Fig. 1f, and the vortex array can also be represented as a displacement map, as shown in Supplementary Fig. 1. Through the SAED, GPA analysis, and polarization map based on HAADF images, we demonstrate a long-range order characterized vortices in the as-grown superlattices.

### Mechanically induced vortex transition

**Fig. 2:**
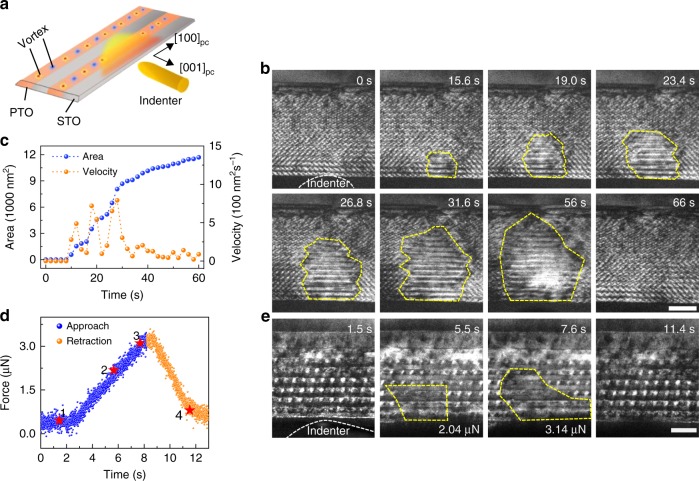
Mechanical manipulation of vortices by in situ transmission electron microscopy (TEM). **a** Schematic image of the experimental setup, with a mobile tungsten tip acting as an indenter for the mechanical manipulation of vortices. **b** Chronological TEM dark-field image series formed by reflection with **ɡ** = (200) _pc_. Under the mechanical loads, vortex contrast gradually disappears. Scale bar, 40 nm. **c** Corresponding transition area (blue line) and switching velocity (orange line) plotted as functions of time. **d** Mechanical loads as a function of time, with the blue points representing the approach branch and orange points corresponding to the retraction branch. The highlighted red stars along with labels 1–4 correspond to images in **e**. **e** Dark-field images showing the vortex evolution under certain measured mechanical stress. Scale bar, 20 nm.

The (PTO)_11_/(STO)_11_ films were then subjected to in situ mechanical stimulation via a scanning probe, as depicted schematically in Fig. 2a. A time series of dark-field images is shown in Fig. 2b, which was selected from the Supplemental Information (Supplementary Movie 1). Tiny particle-like contrast due to the vortex structure is indicated by the **ɡ**-vector (200)_pc_ excited under two-beam imaging (the dark field image under the (002)_pc_ vector is shown in Supplementary Fig. 2). The polarization switching started from the top surface, and the switched area is indicated by the yellow outlines in Fig. 2b. The nucleation area is plotted as a function of time and is shown in Fig. 2c, and the change in the area of the domain can be used to roughly estimate the switching velocity. The irregular shape in the transition area and the highly fluctuating switching velocity are likely due to pinning and/or the inhomogeneous distribution of the strain field caused by the irregular geometry of the tungsten tip. After removal of the applied stress, the domain returns to its original state. The mechanical force required to drive vortex transition is estimated to be a few µN, as shown in Fig. 2d, e. Using the Hertz model^21,24^, the maximum stress, *P*_max_, can be calculated by the formula *P*_max_ = 3 *F*/2π*r*^2^, where *F* is the force and *r* is the contact radius. Given *r* = 50 nm and *F* = 3.14 µN according to the experimental data in Fig. 2e, the resulting stress is ~0.6 GPa. This value is indeed lower compared with the stress reported for conventional *a* or c domains switching in Pb(Zr_0.2_Ti_0.8_)O_3_^21^ or BaTiO_3_ films^25^, indicating that the transition of vortices is less energy-consuming and may be feasible for device applications.

### Phase-field simulation

We used the phase-field method to model triple-layer (PTO)_10_/(STO)_10_ superlattices with an STO layer acting as the top layer exposed to air. Supplementary Fig. 3a–c shows the transformation of polar vortices under a mechanical load. The application of mechanical force causes a change in domain structure from polar vortices (Supplementary Fig. 3a) to in-plane polarization (Supplementary Fig. 3b), in excellent agreement with the experimental observations. As expected, larger applied force magnitudes lead to a greater fraction of the vortex to in-plane polarization changes (Supplementary Fig. 3c).

### Tracking the vortex transition by SAED

**Fig. 3:**
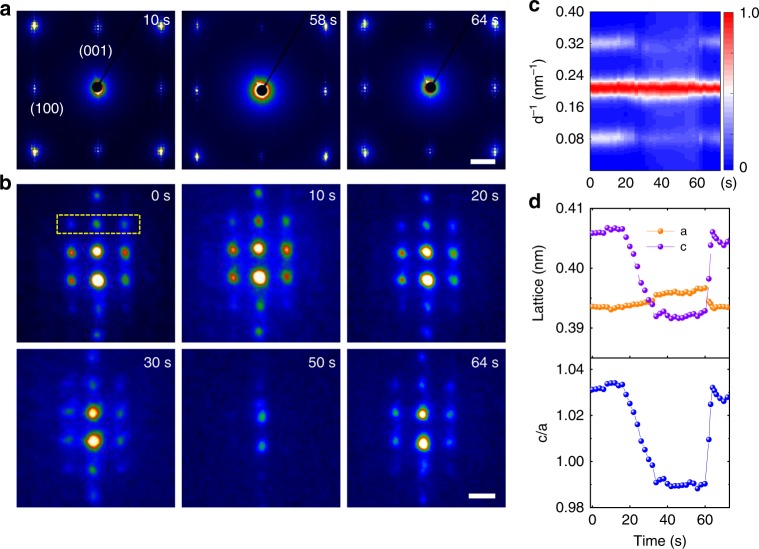
Structural evolution of vortices under mechanical stress. **a** Three SAED images extracted from a real-time image series corresponding to before, during, and after mechanical loading. The vortex reflections disappear at 58 s and recover when the external stress is removed at 64 s. Scale bar, 2 nm^−1^. **b** Chronological SAED (001)_pc_ images with vortex spots dimming as the mechanical force is continuously applied. Yellow box at 0 s indicates diffraction spots used in **c**. Scale bar, 0.1 nm^−1^. **c** Line profile intensity as a function of time normalized by the center superlattice spots. **d** The in-plane *a* and out-of-plane *c* lattice parameters and *c*/*a* ratio as functions of time. The *c*/*a* ratio is eventually less than 1 under a continuously applied mechanical load, indicating that the vortices have transformed to *a* domains.

The mechanical transition of vortices and the reversibility is further confirmed by the SAED patterns, as shown in Fig. 3a (Supplementary Movie 2). At 50 s in Fig. 3b, the reflections belonging to the periodic vortex array along the in-plane direction disappear, while the reflections of the superlattices along the out-of-plane direction are still visible, indicating that the vortex has been switched. Because the intensity is correlated with the number of ordered vortices in the selected area, we plotted the intensity profile of the vortex spots normalized by the superlattice spots (Fig. 3c). This shows a dramatic decrease between 16 and 35 s, and the intensity of superlattice reflections returns to its original value after ~60 s. Hence, it is reasonable to infer that most vortices in the PTO layer have been annihilated under an increasing mechanical stress during 16 s and 35 s in the selected area, and recovered immediately after removal of the stress.

The in-plane lattice parameter *a*, out-of-plane lattice parameter *c*, and their ratio (*c*/*a*) as a function of time were calculated from the electron diffraction pattern and plotted in Fig. 3d. Under mechanical stress, the out-of-plane parameter decreases gradually from ~405 pm after 16 s, reaches a minimum plateau of ~390 pm after 35 s, and returns to its original value at ~60 s as the external stress is removed, whereas the in-plane lattice slightly increases during load application. Therefore, the ratio of the out-of-plane to in-plane lattice parameter decreases from 1.03 to 0.99, indicating that the newly formed phase is the *a* domain. For the pristine vortex state, the smaller in-plane lattice is a result of the continuously lattice rotating of the vortex, while after transition, the uniform *a* domain is formed with a long lattice axis along the in-plane direction, giving rise to a slight increase of the in-plane lattice. The formed *a* domains are supposed to have very different properties, for example, the piezoelectric and nonlinear optical properties can change with order of magnitude due to the transition from vortex to ferroelectric phase^17^. The exotic electrotoroidic^26^, piezotoroidic^27^, and pyrotoroidic^28^ properties that arose from the toroidal moment have also been reported, which are different with those in conventional *a* domains. Our ability to control the transition between vortices and the ferroelectric phase brings to the forefront the possible applications that allow one to largely tune the properties.

### Single-vortex evolution

Single-vortex evolution was captured by the chronological high-resolution TEM image series in Supplementary Fig. 4a (Supplementary Movie 3). The inverse fast Fourier transform (IFFT) image is shown in Supplementary Fig. 4b, through which each vortex can be located. The vortex number decreases with a small time delay between each layer (Supplementary Fig. 5a), implying that for the transition in each PTO layer to occur, there may be a critical stress, which provides the possibility of accurately controlling vortex switching in each layer.

**Fig. 4:**
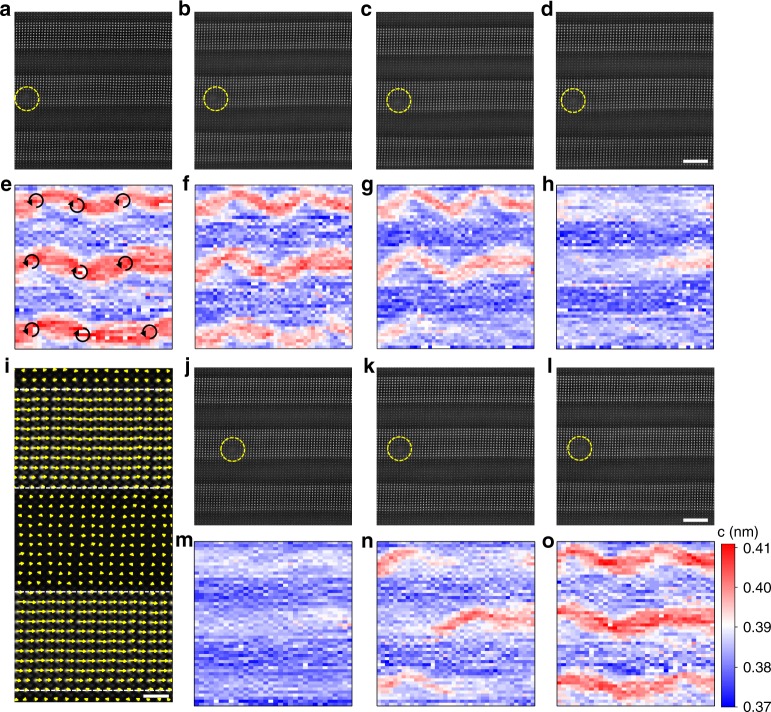
Tracking stress induced vortex transition at the atomic scale. **a**–**d** A series of HAADF-STEM images acquired with applying mechanical loads. Scale bar, 4 nm. **e**–**h** The corresponding out-of-plane lattice mapping of **a**–**d**, showing the transition process. The core of the vortex survives before the transition into a pure *a* domain. The rotation arrows indicate the core positions of each vortex. **i** A high-magnification HAADF-STEM image with the overlaid arrows showing the formed *a* domain after the transition. **j**–**l** HAADF-STEM images acquired during the unloading process. Scale bar, 4 nm. **m**–**o** The corresponding out-of-plane lattice mapping of **j**–**l**, indicating the spontaneous recovery of the vortex after removal of the external mechanical stress. The same scale is used for all the mapping figures. All the HAADF-STEM images were acquired in the same region indicated by the intentionally made marker using the electron probe (see the dashed yellow circles).

To confirm the atomic structure and polarization after disappearance of the vortex, a series of HAADF-STEM images (Fig. 4a–d) was recorded with applying mechanical loading to track the transition at the atomic level in the same marked regions. The sinusoidal lattice waves (Fig. 4e–h) resulted from the continuously lattice rotating that gradually evolved into a uniform contrast, implying that the *a* domain is formed. Low-magnification HAADF-STEM images demonstrated that the boundary denoted by the yellow lines in Supplementary Fig. 6 is sawtooth-shaped. This is further indicative that the switched domain is the *a* domain, because for *a* domains, the inclined (110)-oriented domain walls have the lowest energy, whereas for *c* domains, the vertical (100)-oriented domain walls are the most stable ones^29^. From the HAADF image, the polarization vector map can be extracted from the off-center displacements between Pb and Ti as shown in Fig. 4i, which further confirms that the polarization is in-plane in the switched area. An inhomogeneous recovery behavior was also revealed at the atomic level in Fig. 4j–o.

### Vortex stability under mechanical loading

Although the vortices can be annihilated and recovered, they are not mobile during mechanical loading, based on the high-resolution TEM and atomically resolved HAADF images. To determine the stability of the vortex, we selected the third layer PTO in Supplementary Fig. 4a, and determined the positions of the vortices by IFFT. The trajectory is plotted in Supplementary Fig. 5b, and the lines at 9.0 and 11.1 s are straight and overlapped. The vortex disappears in the next 150 ms after a total of 11.1 s, with no movement observed in our experiment. Owing to a series of HAADF images in Supplementary Fig. 6a, the stability of vortices can be further confirmed. The positions of the vortices determined by these GPA images in Supplementary Fig. 6c (see “Methods”) do not change at all within the uncertainty limits of the experiment. This stability is ideal for data storage because it will reduce the incidence of missing data or cross-talk.

## Discussion

The atomically resolved images during switching indicated that there may be a critical value of stress to switch a vortex. In Supplementary Fig. 7, the vortex stably sustains at 10.95 s. The switching seems to be initiated on the periphery of the vortex and then pushed forward to the inner vortex core (Supplementary Fig. 7). As the core is the most distorted part, it requires a large amount of energy derived from the mechanical stress to overcome the barrier in Landau energy and break the distorted vortex core. Once the core is broken, the entire vortex is quenched quickly within 150 ms. The sinusoidal out-of-plane lattice shrank under the applied mechanical stress, indicating the decrease of the lattice c and the occurrence of the transition, while the core of the vortex is survived before transition into a uniform *a* domain (Fig. 4a–h), which further confirms that the vortex is broken down from the outer part to the inner core. This behavior is different from that proposed in previous theoretical work^30^, whereby vortices were predicted to move and melt to reduce the electrostatic energy under an electric field. In contrast, in our case, the compressive out-of-plane stress drives the rotation of polarization toward the in-plane direction, leading to a reduction in the elastic energy. After retraction of the external stress, the vortices are recovered spontaneously (Fig. 4j–o) because the boundary conditions return to the original state and drive the formation of vortices. Hence, our experiment shows that by modulating the mechanical load, we can control the phase-transition process and manipulate the electric toroidal order on the nanometer scale.

It should be pointed out that such phase-transition process can be very fast based on previous studies. Theoretical simulations^17,31^ predicted that the domain evolution and polarization reversal can be finished within tens of picoseconds. Experimentally, a vortex domain was found to switch on a 100-ns timescale on circular ferroelectric capacitors^32^. In Fig. 4, we intentionally controlled the mechanical loads as a slow ramp in order to track the details at the atomic level similar to previous electrical switching^33^. In another switching event in Supplementary Fig. 8, we find that a relatively large loading results in a very quick transition process that exceeds the temporary resolution of the camera (0.04 s), further confirming that the switching time really depends on the loading of mechanical stress and switching can be very fast.

The contribution of the flexoelectric effect on the vortex transition can also be revealed by phase-field simulations. Previous study^25^ reported that the probe-induced flexoelectric effect prefers to form the *c* domain with downward polarization. Thus, the generated *a* domain under the mechanical loads indicates that the flexoelectric effect does not dominate the transition in this study. In fact, the simulation (Supplementary Fig. 9) shows that the flexoelectric effect does stabilize the parallel *a* domains that are observed in the experiment. In contrast, the antiparallel *a* domains that are thermodynamically equivalent states are generated if the flexoelectric effects are not considered.

The phase-field simulation also suggests that the STO layer is weakly polarized with mean magnitude ~0.063 Cm^−2^ for pristine state and ~0.054 Cm^−2^ after switching as shown in Supplementary Fig. 10. Direct experimental measurement of such small polarization, however, is very challenging even for the previous static electron microscopy study^23^. It becomes much more difficult for our in situ study that generally suffers larger instabilities such as vibration and drift. Nevertheless, the evolution of in-plane lattice *a* and out-of-plane lattice c of STO is extracted from Fig. 4 in Supplementary Fig. 11, from which the lattice parameters essentially keep unchanged during switching (Supplementary Fig. 12), indicating no substantial change in the polarization of STO, which is in agreement with the simulation results (Supplementary Fig. 10).

Previous seminal work^1^ has clarified the feasibility of a new device by making use of the toroidal vortices since the electric field inside the vortex is nonuniform due to the extinct polarization configurations^34^. The PTO/STO superlattice systems are superior to data storages because the vortices inside them have a long-range character, which would otherwise require an arranged arrays of nanodots, nanocomposites, or nanowires to construct a vortex array. Compared with the unfavorable methods like torque^35^, curl electric field^14^, or sweeping electric field^36^ to induce the transition of the toroid order, the mechanical methods we adopted are readily accessible—only an indenter with small mechanical force is needed. Furthermore, the mechanical force would not cause a big fluctuation in temperature, while the electric current naturally induces more heat in the devices, which would degenerate the vortices to *a* domains when the temperature went up to ~ 473 K^17^. The limitation we have is that now the precise quantitative relation between every single vortex in each PTO layer is absent, disabling the accurate control of every single vortex independently in these arrays in the superlattice. To solve this, a more complicated device with delicate mechanical manipulation is needed in the future.

In summary, we performed a systematic study of the dynamics of polar vortices in PTO/STO superlattices under a mechanical stimulus using an in situ (S)TEM technique. Our results demonstrate that vortices transform into *a* domains under mechanical compressive loads and spontaneously return after removal of the external loads at the atomic level. These results provide valuable insights into understanding the fundamental properties of the topological defects in ferroics. The demonstrated ability to control a single vortex by external stress paves the way toward the development of advanced high-density storage devices.

## Methods

### Synthesis of (PbTiO_3_)_11_/(SrTiO_3_)_11_ superlattices

Superlattices consisting of alternating stacks of PbTiO_3_ (PTO) and SrTiO_3_ (STO) layers were deposited on SrRuO_3_ (SRO)-buffered (110)-DyScO_3_ substrates using pulsed-laser deposition (PLD, PVD-5000). A KrF excimer laser (*λ* = 248 nm) with a 10-Hz pulse repetition rate was used to ablate ceramic targets of SrRuO_3_, SrTiO_3_, and Pb_1.1_TiO_3_ for the deposition of the SRO and PTO/STO superlattice sublayers, where the SRO and PTO/STO superlattices were prepared under laser energies of 400 and 360 mJ pulse^−1^, respectively. The SRO-buffered layer was first deposited at a substrate temperature of 690 °C and oxygen pressure of 80 mTorr, and then the substrate was cooled to 600 °C for the alternating deposition of the PTO and STO layers at a 200-mTorr oxygen pressure. The thicknesses of the SRO, PTO, and STO sublayers were controlled to be 30, 11, and 11 unit cells, respectively, based on the deposition time. Selecting the correct laser energy was crucial for ensuring the layer-by-layer growth of the SRO, PTO, and STO sublayers, which is a prerequisite for the presence of polar vortices in the superlattice. Immediately after growth, the (PbTiO_3_)_11_/(SrTiO_3_)_11_ superlattice samples were cooled to room temperature at 50 °C/min at an oxygen pressure of 200 mTorr.

### In situ TEM

In situ TEM experiments were carried out on a JEOL ARM 300 F instrument at 300 kV in either TEM or STEM mode. Experiments were also partly performed in an FEI F20 microscope operated at 200 kV in TEM mode together with a Hysitron system (PI 95) for quantitative measurement of the mechanical loads. However, for Hysitron holder, the stability is too poor to allow us get any atomic resolution because the mechanical force measurement requires feedback that causes relatively larger vibrations. For better stability and resolution, the real-time diffraction patterns, high-resolution TEM images, and atomically resolved STEM images were recorded on a JEOL ARM 300 F with a PicoFemto double-tilt TEM-STM holder provided by ZepTools Technology Company. A tungsten tip acted as an indenter, and was precisely controlled by a piezoelectric system. A STEM image was obtained each time the tungsten tip moved forward for time-lapse STEM images with a convergence angle of 18 mrad and collection angles of 54–220 mrad.

### HAADF-STEM image analysis

An atomically resolved STEM image of the switched area was filtered in Fourier space for polarization determination. Atom positions were determined by a two-dimensional Gaussian peak-fitting method coded in MATLAB. Geometric-phase analysis was performed using the FRWR tools plugin for the Digital Micrograph software. The GPA parameters used for the STEM image series were all the same. IFFT images were acquired by selecting the spots belonging to vortices, and the positions of vortices in the IFFT images were determined by circling the most intense region and narrowing the circle to a spot. Supplementary Fig. 6c shows the peak position of the GPA images from Supplementary Fig. 6b. The marker in Fig. 4 was made by a focused electron beam.

### Phase-field simulation

Phase-field modeling is utilized to model the evolution of the ferroelectric superlattice polarization field, where the polarization is obtained by solving the time-dependent Ginzburg–Landau (TDGL) equations

1 $$\frac{{\partial P_i\left( {{\boldsymbol{r}},t} \right)}}{{\partial t}} = - L\frac{{\delta F}}{{\delta P_i\left( {{\boldsymbol{r}},t} \right)}},\left( {i = 1,2,3} \right)$$

where *L*, ***r***, and *t* represent the kinetic coefficient, spatial vector, and time. The polarization field is represented by *P*_*i*_ and the free energy functional is represented by *F* and contains electric (electrostatic), elastic, Landau bulk, and gradient energies:

2 $$F = {\int} {\left( {f_{{\mathrm{Landau}}} + f_{{\mathrm{elastic}}} + f_{{\mathrm{electric}}} + f_{{\mathrm{flexoelectric}}} + f_{{\mathrm{gradient}}}} \right)} dV$$

The exact expressions and material properties of each energy contribution can be found in the literature^37–40^. The flexoelectric contribution is described by

3 $$f_{{\mathrm{flexoelectric}}} = - \frac{1}{2}f_{ijkl}\left( {P_k\frac{{\partial u_{ij}}}{{\partial x_l}} - u_{ij}\frac{{\partial P_k}}{{\partial x_l}}} \right)$$

where *f*_*ijkl*_ is the flexoelectric coupling tensor, *u*_*ij*_ is the strain field tensor, *x*_*i*_ is the *i*^th^ component of the spatial position vector, and *P*_*k*_ is the *k*^th^ component of the polarization vector. The flexoelectric coupling coefficients were taken from literature^41^.

Arising from the inhomogeneity in elastic constants in the (PbTiO_3_)_11_/(SrTiO_3_)_11_ superlattices, a spectral iterative perturbation method was utilized to solve the mechanical equilibrium equation and thus provide the elastic energy contribution^42^. The lattice constants of SrTiO_3_ and PbTiO_3_ are taken from the literature^39,40^, and the lattice parameters for DyScO_3_ were utilized from literature^43^.

The system size of the simulations was taken to be (200 Δ*x*) × (200 Δ*y*) × (120 Δ*z*) where Δ*x* = Δ*y* = Δ*z* = 0.5 nm, and Δ*x,* Δ*y,* and Δ*z* are represented in real space. The thickness of the superlattice was divided between substrate (20 Δ*z*), film (94 Δ*z*), and air (6 Δ*z*). Random noise was utilized to initiate the simulation process.

All phase-field visualizations were created using the Python programming language, NumPy numerical library^44^ to calculate the curl of the vector field, and the Matplotlib library for numerical plotting^45^.

## Supplementary information

Supplementary Information

Description of Additional Supplementary Files

Supplementary Movie 1

Supplementary Movie 2

Supplementary Movie 3

## Data Availability

The data that support the plots within this paper and other findings of this study are available from the corresponding author upon reasonable request.
