# Supplementary material for: Atomic imaging of mechanically induced topological transition of ferroelectric vortices
Source: Nat Commun. 2020 Apr 15;11:1840. doi: 10.1038/s41467-020-15616-y (PMC7160157; doi:10.1038/s41467-020-15616-y)
Supplement: Supplementary file 1 — Supplementary Information [file 41467_2020_15616_MOESM1_ESM.pdf]

Supplementary Information for

Atomic Imaging of Mechanically Induced Topological Transition of Ferroelectric Vortices

Chen et al.

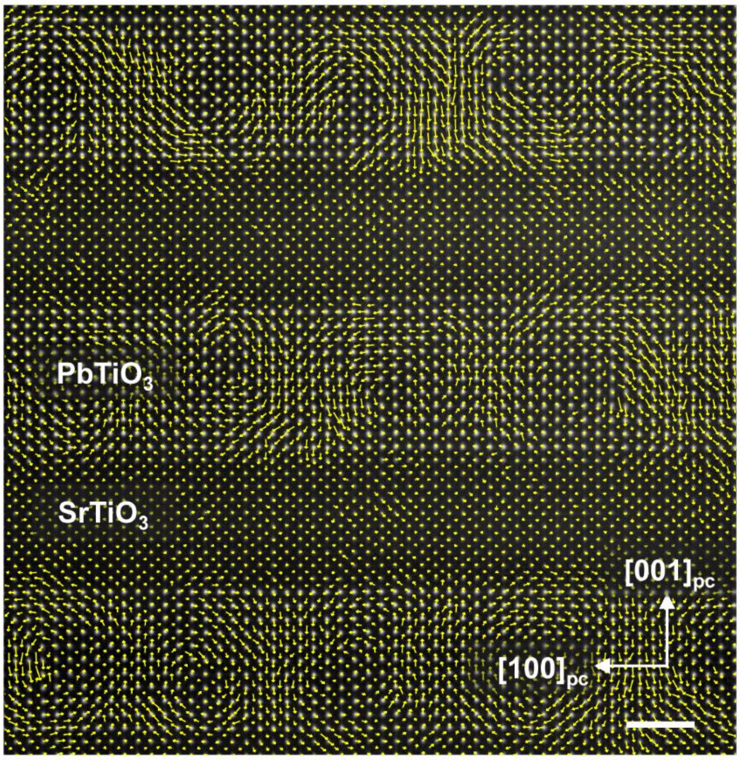

Supplementary Figure 1 Displacement vector map of the $PbTiO_3$/$SrTiO_3$ (PTO/STO) superlattices. Polar displacement map obtained from a high-angle annular dark-field scanning transmission electron microscopy (HAADF-STEM) image showing the clockwise and anti-clockwise polar vortex array[1]. Scale bar, 2 nm.

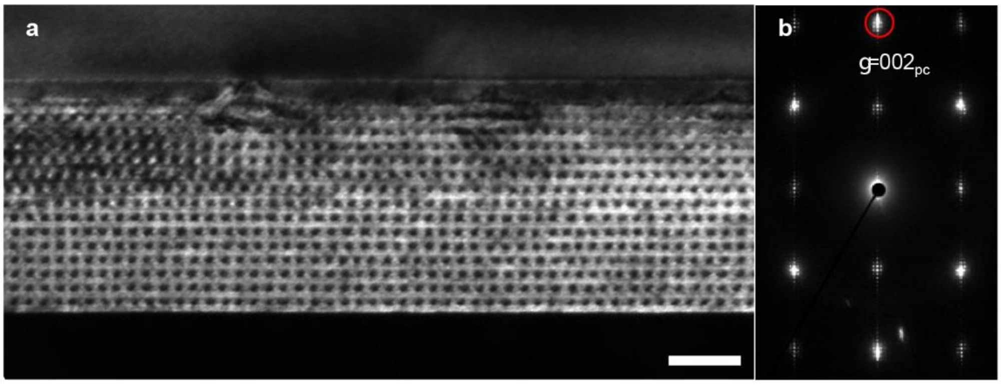

Supplementary Figure 2 Vortex array shown by dark-field image. **a** A dark-field image of PTO/STO with alternating bright and dark contrast. Scale bar, 40 nm. **b** A diffraction pattern of the supperlattices with the red circle indicating the **g** vector used in **a**.

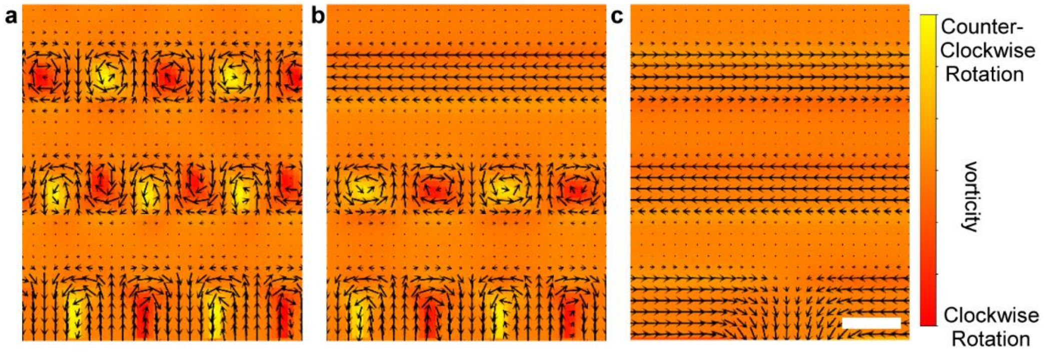

Supplementary Figure 3 Phase field simulation of vortices transition. **a** Simulated initial vortex domain structure in a $(PTO)_{10}/(STO)_{10}$ ferroelectric superlattice, with a thickness of 10 unit cells (UC) in each layer aside from the bottommost PTO layer (12 UC). The top most layer is STO and air. **b** After the application an applied load (~1.5 μN), the domain structure moves toward an in-plane polarization polar state but without complete switching. **c** As a larger load (~5.5 μN) is applied, the domain structure switches to completely in-plane. Scale bar, 4 nm.

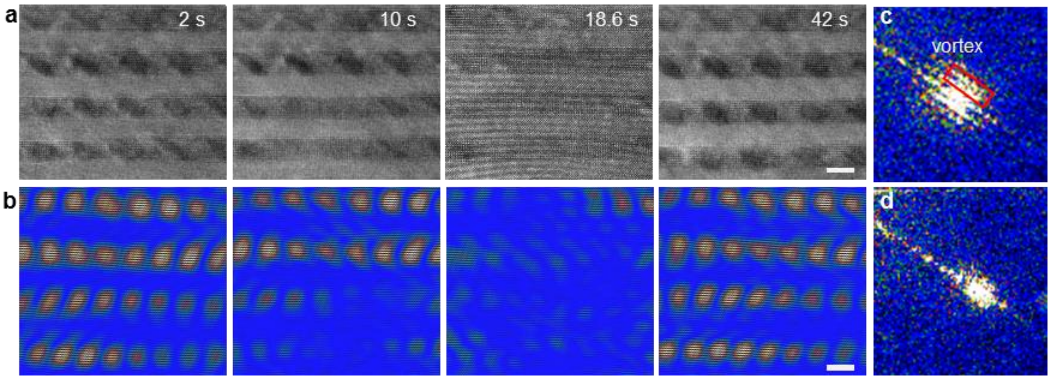

Supplementary Figure 4 Vortex transformation exhibited by inverse fast Fourier transform (IFFT) and fast Fourier transform (FFT) images. **a** A chronological high resolution TEM image series acquired under mechanical loads. Scale bar, 5 nm. **b** The IFFT images of HR-TEM images. **c** $(002)_{pc}$ spots extracted from the FFT of the pristine state with white arrows indicating the additional reflections belonged to the vortices. Scale bar, 5 nm. **d** The FFT image of $(002)_{pc}$ spots after transition. Compare **c** with **d**, it is clear that vortices disappear after mechanical stress.

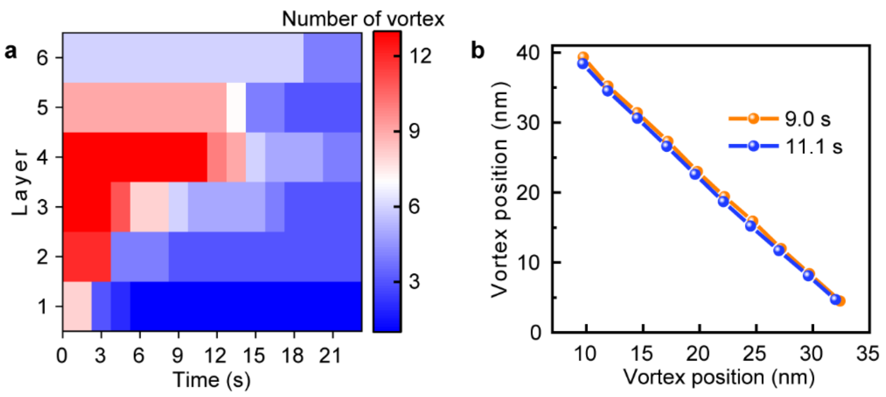

Supplementary Figure 5 The evolution of vortex number and positions by IFFT. **a** The vortex number in each layer as a function of time with time interval of 1.5 s. The vortex number can be clearly counted by the IFFT images as shown in Supplementary Fig. 4. **b** The vortex positions obtained from the IFFT images in 9.0 s and 11.1 s, the latter of which is 150 ms before the vortex vanishing.

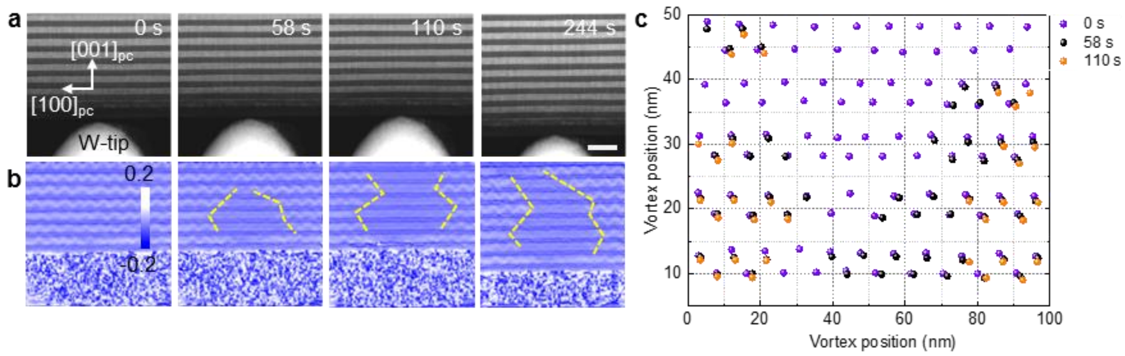

Supplementary Figure 6 Atomic-scale tracking of the vortex transition. **a, b** HAADF-STEM image series acquired under a mechanical load and the corresponding geometric phase analysis (GPA) images, respectively. Scale bar, 20 nm. The yellow lines in **b** show the boundary between the transformed and untransformed areas. **c** Vortex core positons extracted from **b** by locating the position of the peaks in GPA images. The violet, black and orange spots denote the core coordinates of vortices with no, smaller and bigger mechanical force by controlling the displacement of the indenter. Note some of the vortices are transformed in 58 s and 110 s so that their coordinates are missing. Comparing the coordinates of the 'survived' vortices with those of 0 s, it is reasonable to say vortices are stable after transition.

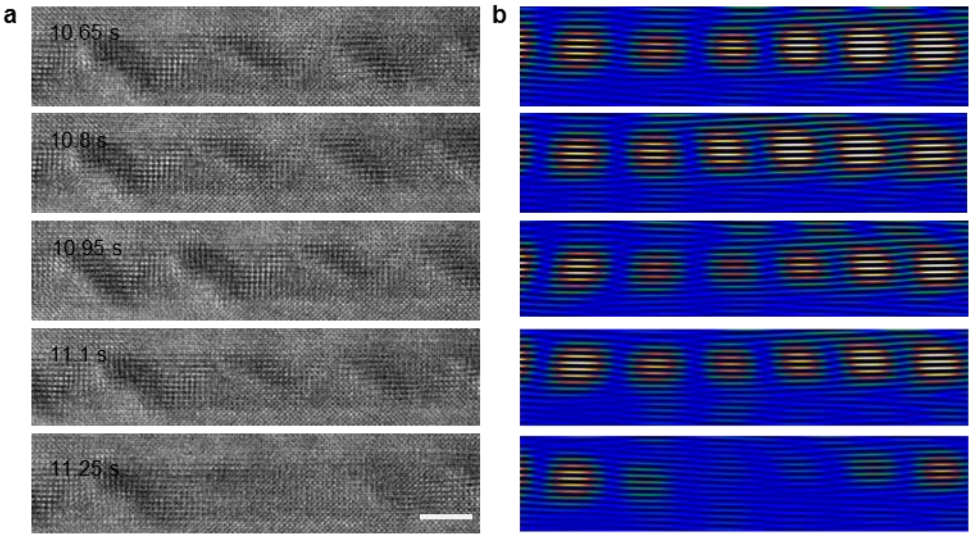

Supplementary Figure 7 Tracking the evolution of vortices by high-resolution TEM images. **a** A clip of TEM images showing the whole transition process of the vortices with time interval of 150 ms. Scale bar, 4 nm. **b** The IFFT images of **a**. Comparing the images in 11.1 s with 10.95 s, the lower part of the vortex began to change while the vortex core still persist as we can see the contrast in **b**. In 11.25 s, the vortex core is broken so that no contrast existed in **b**.

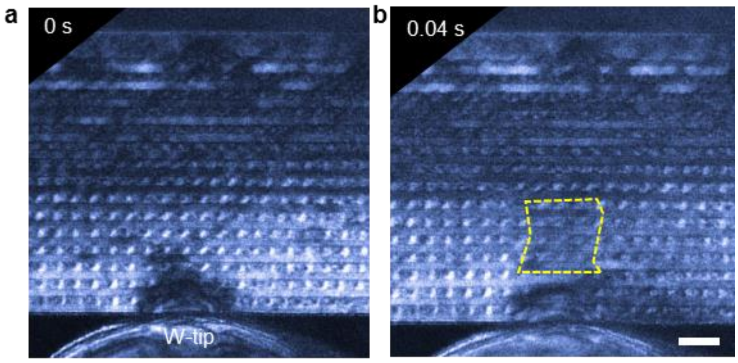

Supplementary Figure 8 Timescale of the vortex transition. **a** Dark-field image showing the vortex before mechanical loading. **b** Vortex transition within 0.04 s (the record limit of the camera) under a large loading. Scale bar, 20 nm.

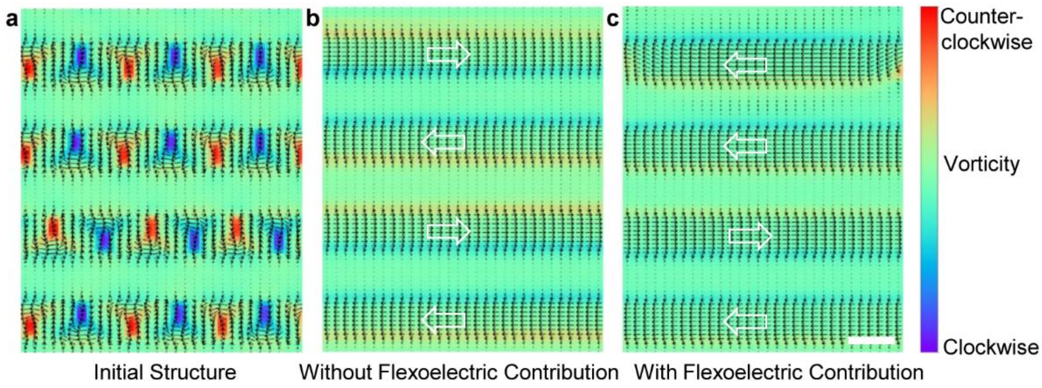

Supplementary Figure 9 The contribution of flexoelectric effect on the vortex transition. **a** The pristine vortex states in the superlattice films. **b** The vortex transitioned into *a* domains which tend to be antiparallel when the flexoelectricity is excluded. **c** The *a* domains in top most PTO layers orient themselves becoming parallel to one another due to the flexoelectric effect. Scale bar, 4 nm.

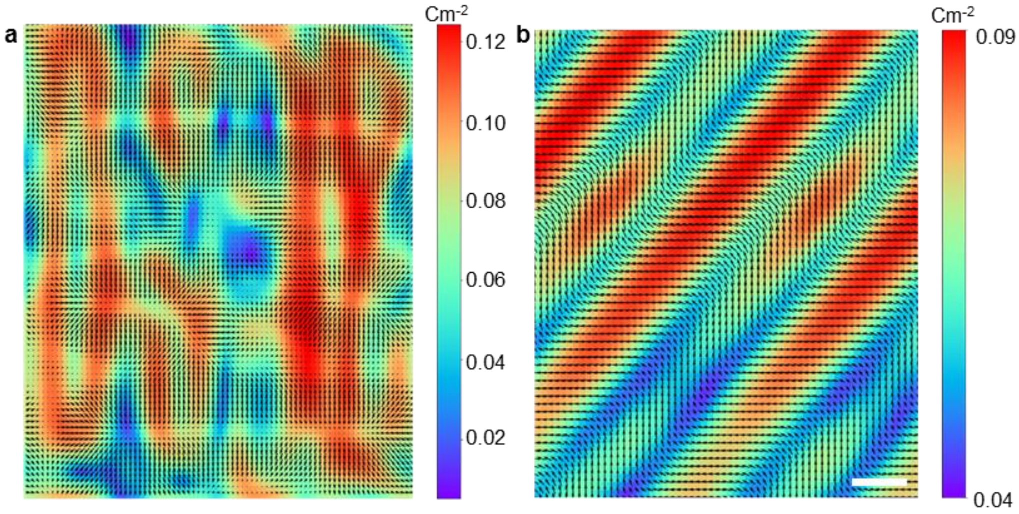

Supplementary Figure 10 The polarization of STO in the *x*-*y* plane before and after mechanical loading. **a** Polarization in the as-grown STO layer. The mean magnitude of polarization is ~0.063 Cm^{-2}. **b** Polarization after mechanical loading. a_1/a_2 domain feature can be recognized while the magnitude is rather small, i.e., the mean polarization ~0.054 Cm^{-2}. Scale bar, 4 nm.

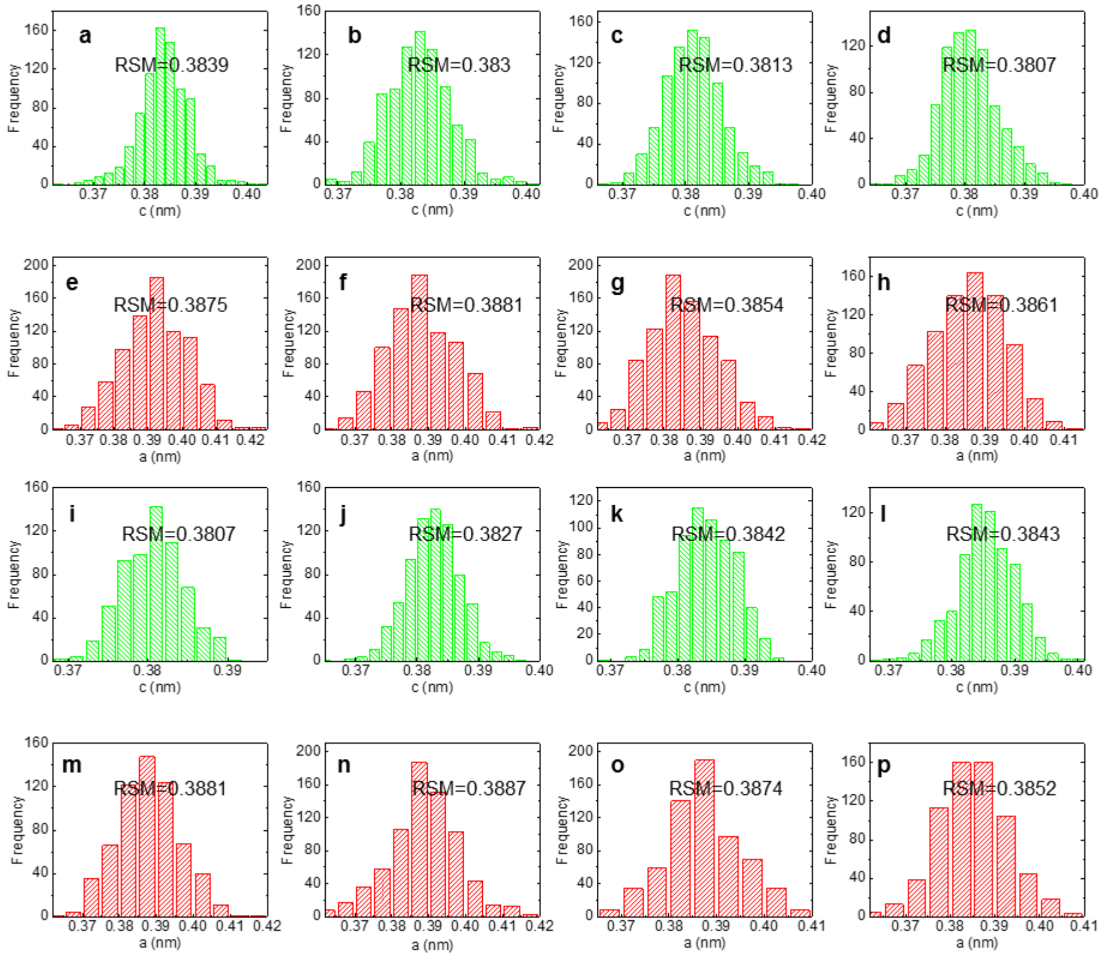

Supplementary Figure 11 Characterization of STO during vortex transition. **a-h** Statistics of the STO out-of-plane **a-d** and in-plane **e-h** lattice calculated on the mechanical loading process based on the HAADF-STEM images in Fig. 4a-d. **i-p** Corresponding out-of-plane **i-l** and in-plane **m-p** lattice on the recovery of vortex process acquired on the HAADF-STEM images in Fig. 4j-l (one HAADF-STEM image is not shown in Fig. 4). The Root Mean Square (RMS) value is labelled in all figures. It should be noted that the measured values are not calibrated by the substrate.

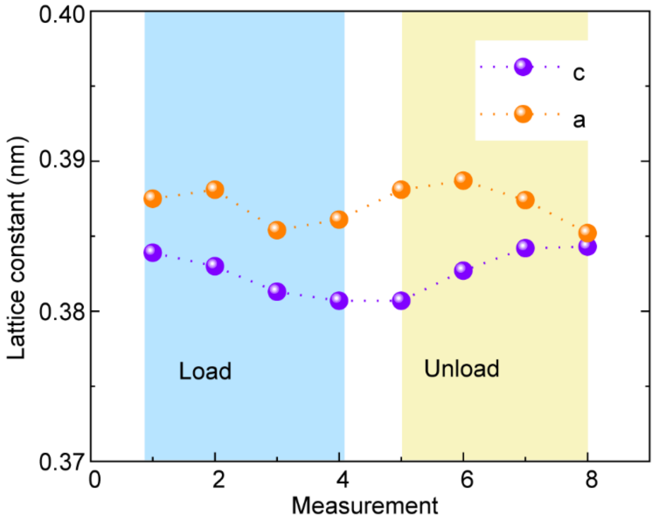

Supplementary Figure 12 The lattice constant of STO. The Root Mean Square (RMS) of the in-plane lattice and out-of-plane lattice during the transition and recovery of the vortex. It has been reported that STO can be polarized by epitaxial strain exert by the substrate such as $DyScO_3$[2]. However, the magnitude of the polarization can be rather small as shown in Supplementary Fig. 10a, making direct experimental determination of polarization in STO challenging. The lattice essentially keep the same, indicating that the polarization in STO is rather small[3].

References

1 Yadav, A. K. *et al.* Observation of polar vortices in oxide superlattices. *Nature* **530**, 198-201 (2016).

2 Haeni, J. H. *et al.* Room-temperature ferroelectricity in strained $SrTiO_3$. *Nature* **430**, 758-761 (2004).

3 Lu, L. *et al.* Topological defects with distinct dipole configurations in $PbTiO_3$/$SrTiO_3$ multilayer Films. *Phys. Rev. Lett.* **120**, 177601 (2018).
