# Supplementary material for: Atomic imaging of mechanically induced topological transition of ferroelectric vortices
Source: Nat Commun. 2020 Apr 15;11:1840. doi: 10.1038/s41467-020-15616-y (PMC7160157; doi:10.1038/s41467-020-15616-y)
Supplement: Supplementary file 2 — Description of Additional Supplementary Files [file 41467_2020_15616_MOESM2_ESM.docx]

**Description of Additional Supplementary Files**

**Supplementary Movie 1.** An in-situ TEM dark-field movie showing the evolution of vortices under the mechanical loads. The particle-like contrast disappears under the indenter. As the mechanical force increased, the area with uniform contrast expended in an inhomogeneous way. The video was recorded at 25 frame/s.

**Supplementary Movie 2.** An in-situ TEM movie showing the diffraction pattern evolution. The additional spots belonged to vortices become dimming and even disappeared, implying the annihilation of vortices. The video was recorded at 5 frame/s.

**Supplementary Movie 3.** An in-situ high resolution TEM movie presenting the transition process of vortices. The video was recorded at 6.6 frame/s.
